# Predictors and moderators of outcome of psychotherapeutic interventions for mental disorders in adolescents and young adults: protocol for systematic reviews

**DOI:** 10.1186/s13643-021-01788-1

**Published:** 2021-08-30

**Authors:** Eleni Vousoura, Vera Gergov, Bogdan Tudor Tulbure, Nigel Camilleri, Andrea Saliba, LuisJoaquin Garcia-Lopez, Ioana R. Podina, Tamara Prevendar, Henriette Löffler-Stastka, Giuseppe Augusto Chiarenza, Martin Debbané, Silvana Markovska-Simoska, Branka Milic, Sandra Torres, Randi Ulberg, Stig Poulsen

**Affiliations:** 1 Department of Psychology, American College of Greece – Deree, 6 Gravias Street GR-153 42 Aghia Paraskevi, Athens, Greece; 2 First Department of Psychiatry, Medical School, National and Kapodistrian University of Athens, Eginition Hospital, 74 Vas. Sofias Ave, 11528 Athens, Greece; 3 Department of Psychology and Logopedics, Faculty of Medicine, University of Helsinki, Helsinki, Finland; 4 West University of Timisoara, Timișoara, Romania; 5 Mental Health Services, Attard, Malta; 6 University of Malta, Msida, Malta; 7 Department of Psychology, University of Jaen, Jaén, Spain; 8 Laboratory of Cognitive Clinical Sciences, Department of Psychology, University of Bucharest, Bucharest, Romania; 9 Sigmund Freud University Vienna, Vienna, Austria; 10 Medizinische Universität Wien, Vienna, Austria; 11 Centro Internazionale Disturbi Di Apprendimento Attenzione E Iperattività, Milan, Italy; 12 Faculty of Psychology and Educational Sciences, University of Geneva, Geneva, Switzerland; 13 Research Department of Clinical, Educational and Health Psychology, University College London, London, UK; 14 Macedonian Academy of Sciences and Arts, Skopje, North Macedonia; 15 Faculty of Psychology and Education Sciences, University of Porto, Porto, Portugal; 16 Institute of Clinical Medicine, University of Oslo, Oslo, Norway; 17 Department of Psychiatry, Diakonhjemmet Hospital, Oslo, Norway; 18 Department of Psychology, University of Copenhagen, Copenhagen, Denmark

**Keywords:** Moderators, Predictors, Psychotherapeutic interventions, Individualized treatment, Young people, Mental health

## Abstract

**Background:**

Adolescence and young adulthood is a risk period for the emergence of mental disorders. There is strong evidence that psychotherapeutic interventions are effective for most mental disorders. However, very little is known about which of the different psychotherapeutic treatment modalities are effective for whom. This large systematic review aims to address this critical gap within the literature on non-specific predictors and moderators of the outcomes of psychotherapeutic interventions among adolescents and young adults with mental disorders.

**Methods:**

The protocol is being reported in accordance with the Preferred Reporting Items for Systematic Review and Meta-Analysis Protocols (PRISMA-P) Statement. PubMed and PsycINFO databases will be searched for randomized controlled and quasi-experimental/naturalistic clinical trials. Risk of bias of all included studies will be assessed by the Mixed Methods Appraisal Tool. The quality of predictor and moderator variables will be also assessed. A narrative synthesis will be conducted for all included studies.

**Discussion:**

This systematic review will strengthen the evidence base on effective mental health interventions for young people, being the first to explore predictors and moderators of outcome of psychotherapeutic interventions for a wide range of mental disorders in young people.

**Systematic review registration:**

PROSPERO CRD42020166756.

**Supplementary Information:**

The online version contains supplementary material available at 10.1186/s13643-021-01788-1.

## Background

The transition from childhood to adulthood is a critical developmental period marked by both risk and resilience [1]. Young people (YP) aged 12 to 30 years are faced with numerous challenges and developmental demands that span across all aspects of their life [2, 3]. Whilst most YP adjust to these new challenges, others struggle and exhibit greater vulnerability for mental disorders. Half of all mental disorders will manifest before the age of 14, and three quarters by the age of 24 [4, 5], making adolescence and young adulthood a critical period for developing mental health disorders.

Studies show that mental health issues impact up to 40% of YP [6, 7], whilst 20% of them formally meet the criteria for functionally impairing mental disorders [8]. Mental and substance use disorders are major contributors to disability in young people worldwide [9–11], accounting for 16% of the global burden of disease and injury in people aged 10–19 years [12]. Moreover, there is strong evidence that mental health disorders in youth, when left untreated, tend to have a recurrent course that persists into adulthood [13]. The consequences of untreated disorders not only impair the individual, but affect society as a whole, through the continuance of psychiatric issues and the increased risk for secondary illnesses resulting in chronic morbidity and mortality, as well as impaired social and work performance [8, 14]. Failure to address mental disorders among YP adequately can have devastating implications for both the present and future, limiting the opportunities of YP to lead healthy and fulfilling lives [12].

The evidence that adolescence and young adulthood constitute a sensitive window of opportunity emphasizes the need to address psychopathology at the population level, maximizing the efficiency, efficacy, and cost-effectiveness of interventions aimed at improving mental health. Untreated mental disorders are discerned as ‘chronic disease’ of the young, leading to persistent and long-term morbidity and mortality [15]. Effective psychotherapeutic treatment improves the course of mental disorders, by reducing the functional impairment and preventing the recurrence of mental disorders [16]. The list of psychotherapeutic interventions two decades ago developed for YP consisted of over 550 treatments [17]. Today, we know that different psychotherapy modalities (cognitive-behavioural, psychodynamic/psychoanalytic, interpersonal, family systems, humanistic/existential, experiential) are generally effective in treating adolescents and young adults [18, 19]. Whilst it has been shown that psychotherapeutic interventions provide some reduction in the burden of mental disorders compared to untreated YP [20, 21], their overall effect on mental health outcomes is moderate [22, 23]. Furthermore, despite significant advances in psychotherapy research in the last decades, research fails to show an improvement of youth psychotherapy outcomes over the years [24, 25]. It is therefore important to investigate and elucidate the factors, which contribute to and influence the outcome of psychotherapy interventions among YP in order to provide more effective and personalized care to this at-risk group [26].

### Predictors and moderators of psychotherapy outcome

There is a growing consensus in the field of mental health that the focus of outcome research should be extended from the overall effectiveness of treatments (i.e. ‘what works in general’) to understanding which clinical factors render a given treatment particularly effective or ineffective (i.e. ‘what works for whom and under what circumstances’) [27]. Such variables that might affect the strength or direction of the treatment response are described as *treatment predictors* and *moderators*. Both predictors and moderators account for the between-individuals variance of treatment outcome [28]. Predictors of treatment outcome are *pre-treatment* variables (although conceptually they can also represent variables measured during treatment) that influence outcome regardless of treatment condition. In other words, they are *prognostic factors* [29] that allow us to determine which patient may benefit most from a given treatment, and vice versa, which patient may respond less optimally or even worsen following a particular treatment. Moderators of treatment outcome, on the other hand, are pre-treatment variables that differentially influence outcome depending on treatment allocation. They reveal which characteristics render a particular treatment more effective than another. Treatment moderators, therefore, represent *prescriptive factors* that guide treatment decisions by for a given patient [29].

Identifying patient and therapist characteristics which may have an influence on clinical outcomes could help design personalized interventions tailored to the unique needs of YP, thus maximizing their efficacy [30]. The findings of this research would provide a guideline for clinicians to help elucidate which YP would respond better to a particular intervention as compared to others, or for whom a particular psychotherapeutic intervention would prove to be inadequate or even ineffective [31]. In terms of policy, identifying these subpopulations of YP might be used to inform service development for health and mental health initiatives. The benefits of identifying treatment predictors and moderators extend to research. Knowing which factors predict differential response to treatment can maximize power in future clinical trials by clarifying the variables on which to stratify and improve validity by guiding the choice of exclusion and inclusion criteria [32]. Conceptually, moderators may help signal differential processes that operate in specific subgroups, generating search for relevant mediator variables and processes, which is a much-needed direction of psychotherapy research.

Personalized medicine is rapidly emerging as a state-of-the-art approach to diagnostics and therapeutics and is beginning to revolutionize our health care systems, promising better treatment for all [33]. In the field of psychotherapy though, personalization of treatment has for long been a fragmented and unsystematic enterprise guided by clinical intuition rather than empirical evidence. In more recent years, advances in computational science have enabled psychotherapy researchers to develop and test methods of optimizing treatment packages for the individual patient [34, 35], although there are inconsistent findings regarding the extent to which personalized treatment has in fact led to improved outcomes [36, 37].

Despite the slow progress in the field of treatment personalization, there is no doubt that a systematic synthesis of treatment predictors and moderators is the first necessary step towards tailoring treatments for each person to maximize effectiveness [28]. And whilst substantial ground has been covered in the adult literature [38–40], this is not the case in treatment for mental disorders in adolescents and young adults [30]. What patient characteristics (predictors/moderators, e.g. gender, level of interpersonal problems, diagnosis) interfere with specific treatment techniques in youth psychotherapy remains largely unknown. The majority of the scarce systematic reviews with YP have been conducted on anxiety and depressive disorders [41–43], followed by non-suicidal self-injury [44], paediatric obsessive–compulsive disorders [45], anorexia and bulimia nervosa [46], and autism spectrum disorder [47]. Whilst some clinical, sociodemographic, and parental/familial variables have emerged as putative prognostic factors, the evidence is inconsistent and limited by underpowered and flawed research designs. Few studies have investigated transdiagnostic predictor and moderator variables of treatment outcome [e.g. 42], and several disorder groups have not yet been investigated for treatment predictors/moderators. Moreover, to our knowledge, no existing review has attempted to capture the entire developmental period from adolescence to young adulthood for a wide range of psychological disorders. Thus, there is an empirical knowledge gap on personalized mental health treatment for YP, which makes the goals for individualized treatment hard to reach [30, 48].

Recognizing this important gap in the literature, the European Cooperation in Science and Technology (COST) funded a 4-year program for the creation of a European Network on Individualised Psychotherapy Treatment of Young People with Mental Disorders (TREATme; https://www.treat-me.eu/). The main aim of the TREATme COST action is to create a European multidisciplinary researcher network focusing on stratification tools to individualize psychotherapy for YP with mental disorders. One of the deliverables of this COST action is to conduct a review of the current literature within the field, so to elucidate putative specific markers of changes in different therapeutic modalities.

The overarching aim of the proposed study is to carry out a number of systematic reviews of the published literature on predictors and moderators of the outcomes of psychotherapeutic interventions in YP with mental disorders. The objectives of this review are to (1) to identify predictors and moderators of psychotherapy treatment outcome for a variety of mental disorders among adolescents and young adults and (2) to evaluate the proportion of clinical trials adopting robust methodological practices in the assessment of these predictors and moderators.

## Methods

### Search strategy

The review was written following the PRISMA for protocols guidelines (PRISMA-P [49]) checklist. The population, intervention, comparison, outcome, and study design (PICOS) strategy [50] was used to specify the research question and guided the forming of the search string for this systematic review (see Table 1 for full PICOS strategy). PubMed and PsycINFO electronic databases were used for a literature search until April 22, 2021. The protocol has been registered at The International Prospective Register of Systematic Reviews (PROSPERO) with the registration number CRD42020166756.

**Table 1:** PICOS and inclusion and exclusion criteria

PICOS strategy	Include	Exclude
P—population	Adolescents (12–18 years) and young adults (19–30 years) with a mental disorder diagnosis: anxiety, obsessive–compulsive and trauma-related disorders, depressive and bipolar disorders, psychotic disorders, eating disorders, personality disorders, substance-related disorders, autism, attention deficit/ hyperactivity disorder, conduct disorders Filters: adolescent OR young adult Keywords: **Anxiety Disorders:** anxiety disorder; neurotic disorder; panic disorder; agoraphobia; social phobia; social anxiety; mutism; separation anxiety; phobic disorder; phobia; generalized anxiety; obsessive compulsive; ocd; hoarding; body dysmorphic disorder; body image disorder; trichotillomania; hair pulling disorder; excoriation disorder; dermatillomania; skin picking disorder; trauma and stressor related disorders; traumatic stress disorder; posttraumatic stress disorder; stress disorder, post-traumatic; ptsd; acute stress disorder; adjustment disorder; **Depressive Disorders:** mood disorder; depressive disorder; depression; affective disorder; dysthymic disorder; dysthymia; premenstrual dysphoric disorder; seasonal affective. **Bipolar Disorders:** bipolar and related disorders; bipolar disorder; mania; manic depression; bipolar depression; pediatric bipolar; cyclothymic disorder; cyclothymia. **Psychotic disorders:** schizophrenia spectrum and other psychotic disorders; psychotic disorder; psychosis; psychoses; schizophrenia; schizoaffective; schizophreniform; reactive psychosis; reactive psychoses. **Eating disorders:** feeding and eating disorder; feeding disorder; eating disorder; anorexia; bulimia; binge eating; pica; rumination disorder; avoidant restrictive food intake; arfid; avoidant eating; purging disorder; night eating syndrome; food addiction; orthorexia; ednos; ofsed. **Personality disorders:** personality disorder, schizotypal personality, schizoid personality; paranoid personality; narcissistic personality; borderline personality; histrionic personality; antisocial personality; obsessive compulsive personality; avoidant personality; dependent personality; character pathology; character neurosis; axis II disorder. **Substance use disorders:** substance related disorder; substance use disorder; substance abuse; substance misuse; substance dependence; addiction; drug use; drug abuse; drug addiction; alcohol related disorder; alcohol use disorder; alcohol abuse; alcohol dependence; alcoholism; amphetamine; cocaine; inhalant; marijuana; cannabis; opioid; heroin; opium; morphine; hallucinogen; tobacco; nicotine; smoking; polydrug; stimulant; substance induced psychosis; substance induced psychotic disorder; drug psychosis; drug psychoses. **Autism**: autistic spectrum disorder; autism spectrum disorder; autistic disorder; autism; asperger syndrome; asperger; asperger’s; child development disorders, pervasive; pervasive child development disorder. **ADHD**: attention deficit disorder; adhd; hyperkinetic disorder; attention deficit hyperactivity disorder. **Conduct disorders:** conduct; conduct disorder; oppositional defiant; defiant disorder; externalizing behavior; externalizing behavior; antisocial behavior; antisocial behaviour	Age range or mean age of participants under 12 or over 30 years. Participants not being diagnosed or having disorder-specific symptoms below the agreed-upon cut-off point.
I—intervention	Psychotherapeutic interventions: Keywords: Psychotherapy; Psychotherapeutic treatment; Psychotherapeutic intervention; Psychological therapy; Psychological treatment; Psychological intervention; Psychosocial therapy; Psychosocial treatment; Psychosocial intervention; Supportive therapy Supportive treatment; Counselling; Counseling; Motivational interviewing; Psychoeducation; Psychoeducational; Cognitive therapy; Cognitive analytic therapy; Behavioral therapy; Behavioural therapy; CBT; Psychoanalysis; Psychodynamic therapy; Psychoanalytic therapy; Dynamic therapy; Transference focused (therapy); Mentalization based (therapy); Metacognitive therapy; Interpersonal therapy; Interpersonal and social rhythm therapy; Schema therapy; Schema-focused therapy; Acceptance and Commitment Therapy; Acceptance based (therapy); Problem solving therapy; Problem solving treatment; Insight oriented therapy; Rational emotive; Solution focused therapy; Family therapy Family systems therapy; Parenting intervention; Parent management training; Group therapy; Mind–Body Therapy; Art Therapy; Dance Therapy; Music Therapy; Play Therapy; Expressive therapy; Cognitive remediation; Cognitive training; Behavioral activation; Behavioural activation; Behavior activation; Behavioral weight control; Behavioural weight control; Applied behavior analysis; Applied behaviour analysis; Attention bias modification; Exposure and response prevention; Exposure therapy; Systematic Desensitization; Eye movement desensitization reprocessing; EMDR; Psychology biofeedback; Hypnosis; Mindfulness; Relaxation	Prevention programs. Studies testing interventions using only medication arms. Studies with interventions targeting only parents.
C—comparison		No exclusion criteria.
O—outcome	Quantitative studies including pre- and post-measurement published in peer-review journals	Qualitative studies. Dissertations. Book chapters.
S—study design	Clinical outcome trials: RCT, controlled trials, empirical trials, naturalistic setting, case studies Filters: Clinical Trial OR Comparative study	Case studies where *n* < 10 or results not being reported in group level.

Searches were conducted combining search strings for (a) psychotherapeutic intervention search terms, (b) mental disorder search terms, (c) age range search terms, and (d) study type terms. Controlled descriptors (i.e. PubMed MeSH terms, PsycINFO thesaurus) and their synonyms (keywords) were verified in each database. The search terms were combined using the Boolean operators ‘AND’ and ‘OR’.

Searches were conducted separately for each mental disorder. The following mental disorder categories commonly reported among YP were chosen for inclusion: (a) anxiety, obsessive–compulsive and trauma-related disorders; (b) depressive and bipolar disorders; (c) psychotic disorders; (d) eating disorders; (e) personality disorders; (f) substance-related disorders; (g) autism spectrum disorders; (h) attention deficit/hyperactivity disorder; and (i) conduct disorders.

To ensure successful identification of relevant studies for the specific age group targeted, we added age filters for ‘adolescents’ and ‘young adults’. To identify clinical trials, we used the filter for study type, including ‘clinical study’ OR ‘comparative study’ in PubMed and ‘clinical case study’ OR ‘clinical trial’ OR ‘empirical study’ OR ‘treatment outcome’ in PsycINFO. Preliminary manual searches that were carried out with relevant search terms (clinical trial treatment response, treatment outcome, random allocation, controlled trial, efficacy, effectiveness) yielded comparable results to the filters selected. This was used to determine that the chosen filters had adequate sensitivity.

Capitalizing on the culturally diverse background of our research team, we had no a priori restriction regarding the language of the published full text, in order to increase the yield of appropriate articles and, thus, the generalizability of our findings. However, it was decided that the title and abstract must be available in English to be searchable by English keywords and for all team members to be able to appraise the study design.

One researcher (VG) formed the final search strings in collaboration with information specialists and conducted the searches. Two researchers (EV and SP) performed the searches independently to cross-check the results. The open access bibliographic software Mendeley was used to store, organize, and manage all the references and ensure a systematic and comprehensive search. Duplicate publications from the database search results were removed. An example of a search strategy for the PubMed database for anxiety disorders is presented in Table 2 and for PsycINFO in Table 3.

**Table 2:** Search strategy for anxiety disorders on PubMed

Search terms for all disorders:
**1. Treatments:**
"psychotherapy"[MeSH Terms] OR "psychotherapy"[All Fields] OR "psychotherapeutic treatment"[All Fields] OR "psychotherapeutic treatments"[All Fields] OR "psycho-therapeutic treatment"[All Fields] OR "psychotherapeutic intervention"[All Fields] OR "psychotherapeutic interventions"[All Fields] OR "psychological therapy"[All Fields] OR "psychological therapies"[All Fields] OR "psychological treatment"[All Fields] OR "psychological treatments"[All Fields] OR "psychological intervention"[All Fields] OR "psychological interventions"[All Fields] OR "psychosocial therapy"[All Fields] OR "psychosocial therapies"[All Fields] OR "psychosocial treatment"[All Fields] OR "psychosocial treatments"[All Fields] OR "psychosocial intervention"[All Fields] OR "psychosocial interventions"[All Fields] OR "supportive therapy"[All Fields] AND "supportive therapies"[All Fields] OR "supportive treatment"[All Fields] OR "supportive treatments"[All Fields] OR "counseling"[MeSH Terms] OR "counselling"[All Fields] OR "counseling"[All Fields] OR "motivational interviewing"[All Fields] OR "psychoeducation"[All Fields] OR "psychoeducational"[All Fields] OR "psycho-education"[All Fields] OR "psycho-educational"[All Fields] OR "cognitive therapy"[All Fields] OR "cognitive therapies"[All Fields] OR "behavioural therapy"[All Fields] OR "behavioural therapies"[All Fields] OR "behavioral therapy"[All Fields] OR "behavioral therapies"[All Fields] OR "cbt"[All Fields] OR "psychoanalysis"[MeSH Terms] OR "psychoanalysis"[All Fields] OR "psychodynamic therapy"[All Fields] OR "psychodynamic therapies"[All Fields] OR "psychoanalytic therapy"[All Fields] OR "psychoanalytic therapies"[All Fields] OR "dynamic therapy"[All Fields] OR "dynamic therapies"[All Fields] OR "transference focused"[All Fields] OR "mentalization based"[All Fields] OR "metacognitive therapy"[All Fields] OR "metacognitive therapies"[All Fields] OR "interpersonal therapy"[All Fields] OR "interpersonal therapies"[All Fields] OR "interpersonal and social rhythm therapy"[All Fields] OR "schema therapy"[All Fields] OR "Schema-focused Therapy"[All Fields] OR "Schema-focused Therapy"[All Fields] OR "acceptance and commitment therapy"[All Fields] OR "acceptance based"[All Fields] OR "problem solving therapy"[All Fields] OR "problem solving therapies"[All Fields] OR "problem solving treatment"[All Fields] OR "problem solving treatments"[All Fields] OR "insight oriented therapy"[All Fields] OR "insight oriented therapies"[All Fields] OR "rational emotive"[All Fields] OR "solution focused therapy"[All Fields] OR "solution focused therapies"[All Fields] OR "family therapy"[All Fields] OR "family therapies"[All Fields] OR "family systems therapy"[All Fields] OR "parenting intervention"[All Fields] OR "parenting interventions"[All Fields] OR "parent management training"[All Fields] OR "group therapy"[All Fields] OR "group therapies"[All Fields] OR "mind–body therapies"[MeSH Terms] OR "mind body therapy"[All Fields] OR "mind body therapies"[All Fields] OR "art therapy"[All Fields] OR "art therapies"[All Fields] OR "dance therapy"[All Fields] OR "dance therapies"[All Fields] OR "music therapy"[All Fields] OR "music therapies"[All Fields] OR "play therapy"[All Fields] OR "play therapies"[All Fields] OR "expressive therapy"[All Fields] OR "expressive therapies"[All Fields] OR "cognitive remediation"[All Fields] OR "cognitive training"[All Fields] OR "behavioral activation"[All Fields] OR "behavior activation"[All Fields] OR "behavioural activation"[All Fields] OR "applied behavior analysis"[All Fields] OR "applied behaviour analysis"[All Fields] OR "behavioral weight control"[All Fields] OR "behavioural weight control"[All Fields] OR "attention bias modification"[All Fields] OR (("attention"[MeSH Terms] OR "attention"[All Fields]) AND bias-modification[All Fields]) OR "exposure and response prevention"[All Fields] OR (exposure[All Fields] AND "response prevention"[All Fields]) OR "exposure therapy"[All Fields] OR "systematic desensitization"[All Fields] OR "eye movement desensitization reprocessing"[All Fields] OR "emdr"[All Fields] OR "psychology biofeedback"[All Fields] OR "hypnosis"[All Fields] OR "mindfulness"[All Fields] OR "relaxation"[MeSH Terms]
**2. Age:**
("adolescent"[MeSH Terms] OR "adolescent"[All Fields] OR "adolescents"[All Fields]) OR ("young adult"[MeSH Terms] OR "young adult"[All Fields] OR "young adults"[All Fields])
**3. Anxiety:**
"anxiety disorders"[MeSH Terms] OR "anxiety disorders"[All Fields] OR "anxiety disorder"[All Fields] OR "neurotic disorders"[MeSH Terms] OR "neurotic disorders"[All Fields] OR "neurotic disorder"[All Fields] OR "panic disorder"[All Fields] OR "panic disorders"[All Fields] OR "agoraphobia"[All Fields] OR "social phobia"[All Fields] OR "social phobias"[All Fields] OR "social anxiety"[All Fields] OR "mutism"[All Fields] OR "separation anxiety"[All Fields] OR "phobic disorders"[All Fields] OR "phobic disorder"[All Fields] OR "phobia"[All Fields] OR "phobias"[All Fields] OR "generalized anxiety"[All Fields] OR ("obsessive–compulsive"[All Fields] AND "disorder"[All Fields]) OR "obsessive compulsive disorder"[All Fields] OR ocd[All Fields] OR "hoarding disorders"[All Fields] OR "hoarding disorder"[All Fields] OR "body dysmorphic disorders"[All Fields] OR "body dysmorphic disorder"[All Fields] OR "body image disorder"[All Fields] OR "trichotillomania"[All Fields] OR "hair pulling"[All Fields] OR excoriation[All Fields] OR dermatillomania[All Fields] OR "skin picking"[All Fields] OR "Trauma and Stressor Related Disorders"[MeSH Terms] OR "traumatic stress disorder"[All Fields] OR "traumatic stress disorders"[All Fields] OR "posttraumatic stress disorder"[All Fields] OR "posttraumatic stress disorders"[All Fields] OR "stress disorders, post-traumatic"[MeSH Terms] OR "post-traumatic stress disorders"[All Fields] OR "ptsd"[All Fields] OR "acute stress disorder"[All Fields] OR "acute stress disorders"[All Fields] OR "adjustment disorders"[All Fields] OR "adjustment disorder"[All Fields]
**4. Filters (Article types):**
Clinical Study[ptyp] OR Comparative Study[ptyp]
**5. Date Filter**
"0001/01/01"[PDAT] : "2021/04/22"[PDAT]

**Table 3:** Search strategy for anxiety disorders on PSycINFO

1. adolescen*.mp
2. young adult*.mp
3. 1 or 2
4. psychotherap*.mp. or exp PSYCHOTHERAPY/
5. Psychotherapeutic treatment*.mp
6. Psychotherapeutic intervention*.mp
7. Psychological therap*.mp
8. Psychological treatment*.mp
9. Psychological intervention*.mp
10. Psychosocial therap*.mp. [mp = title, abstract, heading word, table of contents, key concepts, original title, tests & measures]
11. Psychosocial treatment*.mp
12. Psychosocial intervention*.mp
13. Supportive therap*.mp. [mp = title, abstract, heading word, table of contents, key concepts, original title, tests & measures]
14. Supportive treatment*.mp
15. Counselling.mp
16. exp COUNSELING/ or counseling.mp
17. exp Motivational Interviewing/ or Motivational interviewing.mp
18. exp PSYCHOEDUCATION/ or Psychoeducation*.mp
19. 4 or 5 or 6 or 7 or 8 or 9 or 10 or 11 or 12 or 13 or 14 or 15 or 16 or 17 or 18
20. cognitive therap*.mp. or exp Cognitive Therapy/
21. Cognitive analytic therap*.mp. [mp = title, abstract, heading word, table of contents, key concepts, original title, tests & measures]
22. Behavioral therap*.mp. or exp Cognitive Behavior Therapy/
23. Behavioural therap*.mp. [mp = title, abstract, heading word, table of contents, key concepts, original title, tests & measures]
24. CBT.mp
25. Psychoanalysis.mp. or exp PSYCHOANALYSIS/
26. Psychodynamic therap*.mp
27. Psychoanalytic therap*.mp
28. Dynamic therap*.mp. [mp = title, abstract, heading word, table of contents, key concepts, original title, tests & measures]
29. Transference focused.mp
30. Mentalization based.mp
31. Metacognitive therap*.mp. [mp = title, abstract, heading word, table of contents, key concepts, original title, tests & measures]
32. Interpersonal therap*.mp. or exp Interpersonal Psychotherapy/
33. (Interpersonal and social rhythm therap*).mp. [mp = title, abstract, heading word, table of contents, key concepts, original title, tests & measures]
34. exp Schema Therapy/ or Schema therap*.mp
35. Schema-focused therap*.mp. [mp = title, abstract, heading word, table of contents, key concepts, original title, tests & measures]
36. "Acceptance and Commitment Therap*".mp. or exp "Acceptance and Commitment Therapy"/
37. Acceptance based.mp
38. Problem solving therap*.mp. [mp = title, abstract, heading word, table of contents, key concepts, original title, tests & measures]
39. Problem solving treatment*.mp. [mp = title, abstract, heading word, table of contents, key concepts, original title, tests & measures]
40. exp Insight Therapy/ or Insight oriented therap*.mp
41. exp Rational Emotive Behavior Therapy/ or Rational emotive.mp
42. exp Solution Focused Therapy/ or Solution focused.mp
43. Family therap*.mp. or exp Family Therapy/
44. Family systems therap*.mp. [mp = title, abstract, heading word, table of contents, key concepts, original title, tests & measures]
45. exp Family Intervention/ or exp Parent Training/ or Parenting intervention*.mp
46. Parent management training.mp
47. Group therap*.mp. or exp Group Psychotherapy/
48. exp Mind Body Therapy/ or Mind–Body Therap*.mp
49. exp Art Therapy/ or Art Therap*.mp
50. Dance Therap*.mp. or exp Dance Therapy/
51. Music Therap*.mp. or exp Music Therapy/
52. Play Therap*.mp. or exp Creative Arts Therapy/
53. exp Expressive Psychotherapy/ or Expressive therap*.mp
54. 20 or 21 or 22 or 23 or 24 or 25 or 26 or 27 or 28 or 29 or 30 or 31 or 32 or 33 or 34 or 35 or 36 or 37 or 38 or 39 or 40 or 41 or 42 or 43 or 44 or 45 or 46 or 47 or 48 or 49 or 50 or 51 or 52 or 53
55. Cognitive remediation.mp
56. Cognitive training.mp
57. Behavioral activation*.mp
58. Behavioural activation*.mp
59. Behavior activation*.mp
60. Behavioral weight control*.mp
61. Behavioural weight control*.mp
62. exp Behavior Modification/ or exp Behavior Analysis/ or Applied behavior analysis.mp
63. exp Behavior Therapy/ or Applied behaviour analysis.mp
64. Attention bias modification.mp
65. "Exposure and response prevention*".mp
66. exposure therap*.mp. or exp Exposure Therapy/
67. exp Systematic Desensitization Therapy/ or Systematic Desensitization.mp
68. exp Eye Movement Desensitization Therapy/ or Eye movement desensitization reprocessing.mp
69. EMDR.mp
70. Psychology biofeedback.mp
71. Hypnosis.mp. or exp HYPNOSIS/
72. Mindfulness.mp. or exp MINDFULNESS/
73. Relaxation.mp. or exp RELAXATION THERAPY/ or exp RELAXATION/
74. 55 or 56 or 57 or 58 or 59 or 60 or 61 or 62 or 63 or 64 or 65 or 66 or 67 or 68 or 69 or 70 or 71 or 72 or 73
75. 19 or 54 or 74
76. exp Anxiety Disorders/ or anxiety disorder*.mp
77. exp Neurosis/ or neurotic disorder*.mp
78. exp Panic Disorder/ or panic disorder*.mp
79. agoraphobi*.mp. or exp Agoraphobia/
80. social phobi*.mp. or exp Social Phobia/
81. social anxiety.mp. or exp Social Anxiety/
82. exp MUTISM/ or exp ELECTIVE MUTISM/ or mutism.mp
83. separation anxiety.mp. or exp Separation Anxiety/
84. phobic disorder.mp
85. phobi*.mp. or exp PHOBIAS/
86. exp Generalized Anxiety Disorder/ or generalized anxiety.mp
87. exp Obsessive Compulsive Disorder/ or obsessive compulsive.mp
88. ocd.mp
89. hoarding.mp. or exp HOARDING DISORDER/
90. exp Body Dysmorphic Disorder/ or body dysmorphic disorder*.mp
91. exp Body Image Disturbances/ or Body Image Disorder*.mp
92. trichotillomania.mp. or exp TRICHOTILLOMANIA/
93. hair pulling disorder*.mp
94. excoriation disorder*.mp
95. dermatillomania.mp
96. skin picking disorder*.mp
97. "trauma and Stressor Related Disorder*".mp
98. traumatic stress disorder*.mp
99. exp Posttraumatic Stress Disorder/ or posttraumatic stress disorder*.mp
100. stress disorder, post-traumatic.mp
101. ptsd.mp
102. exp Acute Stress Disorder/ or acute stress disorder*.mp
103. exp Adjustment Disorders/ or adjustment disorder*.mp
104. 76 or 77 or 78 or 79 or 80 or 81 or 82 or 83 or 84 or 85 or 86 or 87 or 88 or 89 or 90 or 91 or 92 or 93 or 94 or 95 or 96 or 97 or 98 or 99 or 100 or 101 or 102 or 103
105. 3 and 75 and 104
106. limit 105 to (("0200 clinical case study" or "0300 clinical trial" or "0400 empirical study" or 2100 treatment outcome) and (200 adolescence or 320 young adulthood))

### Study selection criteria

Eligibility of outcome studies was determined with the following criteria, specified by two researchers (BT, VG): (a) clinical outcome study (b) with at least one treatment condition involved being a psychotherapeutic intervention of any length or orientation for (c) adolescents or young adults aged 12–30 years (d) with specified mental disorders (e) as determined by DSM-5, ICD-10, or other diagnostic criteria or high level of symptoms on at least one relevant self-report measure (above the defined cut-off point for that measure), which (f) reports on the relationship between baseline variables and treatment outcome. In addition, the study must be published in a peer-review journal, and at least the title and abstract must be published in English.

Clinical outcome study was defined as any psychotherapy trial that met the WHO definition as ‘any research study that prospectively assigns human participants or groups of humans to one or more health-related interventions to evaluate the effects on health outcomes’. A wide range of research designs were eligible, including randomized controlled designs, quasi-experimental/naturalistic design (i.e. designs in which participants are assigned to the intervention or control arms based on their availability or preference instead of randomized treatment allocation), or an open trial (i.e. non-controlled, pre-post study). Moreover, the clinical study should report at least two assessment points: pre-treatment (compulsory) and post-treatment (compulsory). Follow-up assessment was not compulsory for study inclusion; however, to be considered a follow-up, at least 1 month between post-treatment and follow-up was necessary for the third assessment point.

Psychotherapeutic interventions were defined as psychotherapy, counselling, and other psychosocial interventions conducted in a dyadic, family, or group therapy setting. We included all bona fide psychotherapies [51]; additionally, given that members of the research team have expertise with different disorder groups, a list of additional psychotherapeutic interventions was compiled and approved by the interdisciplinary research team. We decided against including interventions, such as yoga or dramatherapy, unless they were integrated into a broader psychotherapeutic intervention. To allow for a wide representation of psychotherapeutic interventions, we decided to include non-manualized interventions and we made no a priori inclusion decision regarding the training status of psychotherapy providers.

A more detailed summary of the participants, interventions, comparators, and outcomes considered, as well as the type of studies included according to PICOS strategy, is provided in Table 1.

Moderators of treatment outcome were defined as variables (a) measured at baseline that (b) interact with treatment to change outcome for each sub-group, and (c) the interaction is related to outcome in the linear model with or without a main effect. Predictors of treatment outcome were defined as (a) baseline variables that (b) affect outcome (significant main effect only) but do not interact with the allocated intervention. Both terms have been described in the literature under different terminology [27]. To avoid making any assumptions on the definition of relevant predictors and moderators for treatment outcome in advance, and because these terms are generally not included in keywords or abstracts, we will first conduct a systematic search to identify eligible outcome studies, and from that pool of clinical studies, predictor and moderator studies will be searched manually.

In our study, treatment outcome was operationalized as (a) changes in symptom scores or remission/response/recurrence rates (both those pertaining to the main diagnosis and those related to comorbid disorders) or outcomes reflecting patients’ functional status, such as general wellbeing, distress, coping, emotion regulation, and overall patient welfare (e.g. employment success, social interactions, physical activity, etc.). Psychotherapy process measures (e.g. therapeutic alliance), therapy-related outcomes (e.g. compliance to treatment, satisfaction with treatment, credibility of treatment, motivation for treatment), and attendance and drop-out rates were excluded. Assessment of treatment outcome could be conducted at end-of-treatment and/or follow-up, or during treatment (e.g. repeated measures of symptoms used in a ‘slopes-as-outcome’ model; early treatment response measured during treatment).

### Screening procedure

Screening of studies will be conducted using a three-stage screening process. Stage one will include the screening of all titles and abstracts, performed independently by two researchers for each diagnostic group. During stage two, the pair of researchers will independently screen each full-text article. Any discrepancies in the screening of titles/abstracts and full-text articles will be resolved via discussion between the pair until consensus is reached, and when necessary, a third reviewer (senior rater; SP) will be called in adjudicate. In the event that a full-text article is not available in the databases the research team had access to, the researchers will contact the corresponding authors.

In the final, third step, the eligible outcome studies will be screened for predictors and moderators of treatment outcome. The pair of raters will independently screen eligible studies for candidate predictors and moderators and will reach a consensus on which relevant studies should be included. Eligible predictor/moderator variables are to be included in the proposed study, as long as they are assessed pre-randomization and do not change as a response to treatment [52]. All pairs of coders have received extensive training in the identification and extraction of predictors and moderators and all pairs are using the same pre-agreed codebook. To further ensure integrity and accuracy of coding, a random sample will be extracted for each disorder group and two senior raters (SP and EV) will assess the reproducibility of the data.

### Data extraction and coding

Relevant data will be extracted using a data extraction sheet designed for this systematic review. We plan to extract data from the final list of included studies in the following categories:

- (1) Identification of the study, article title, journal title, authors, publication year, host institution of the study (hospital, university, research centre, single institution, multicentre study), country, target population.
- (2) Sample characteristics: sample size, gender; age, race; diagnostic procedures and measures; sample type (i.e. in-patient, out-patient, school, community, or others).
- (3) Methodological characteristics: study design information regarding randomization and control condition, intervention groups and controls; size (control and intervention); and length of follow-up.
- (4) Predictor and moderators: potential predictors and moderators assessed, operationalization and measurement of each candidate variable, direction of relationship between predictor/moderator and outcome, results of predictor and moderation effect analyses.

Predictors and moderator variables will be further coded based on the structure found in Knopp et al. [40] as (a) non-specific *predictors* of outcome, if the main effect of a predictor on outcome was assessed for the sample as a whole; and (b) *moderators*, if the effect of the baseline variable on outcome was assessed through a direct test of the interaction between the baseline variable and the intervention(s). Variables identified through the splitting of the data into groups will be coded as predictors of outcome. Several strategies for such subgroup analysis can be found in the literature (see [40]). One approach includes assessing the main effect of a predictor on outcome only for the treatment or control group. Another approach is to select patients from the treatment and control groups based on a baseline characteristic and compare intervention with control patients within that subgroup (for example, comparing the efficacy of intervention vs. control among male participants). Another possibility is to split the overall sample into two groups and look at the effect of the predictor on outcomes for both groups separately (e.g. splitting the sample into those with and without comorbid mental disorders and assessing outcomes for both groups separately).

For data extraction, a minimum of two independent reviewers will extract and summarize the data from each included study. Two senior coders (SP and EV) will select a random sample of extracted data for quality check. If the outcome data in the original article is unclear, the corresponding author will be contacted via email for clarification.

### Quality assessment

The Mixed Methods Appraisal Tool (MMAT) [53] will be used to estimate the possible sources of bias and to evaluate the overall study quality. The MMAT is a critical appraisal tool developed to evaluate the methodological quality of empirical studies. Initially developed in 2006, the tool has undergone significant revisions in 2018 and has adequate reliability and validity [54]. In its latest version, it includes a total of 25 criteria and 2 screening questions (8), appraising five different categories of study designs: (a) qualitative, (b) randomized controlled trial, (c) non-randomized, (d) quantitative descriptive, and (e) mixed methods studies. Each study design category is evaluated based on five core criteria and each criterion is rated on a scale of ‘yes’, ‘no’, and ‘can’t tell’. The MMAT tool was chosen on the basis that it allows to assess the quality of different types of studies, including qualitative research, randomized controlled trials, non-randomized studies, quantitative descriptive studies, and mixed methods studies. Given the variety of research design methodologies across included studies, the MMAT tool will provide a comprehensive, yet comparable appraisal of risk of bias across studies with different methodologies. Two independent reviewers will perform the risk of bias assessment and disagreements will be discussed and resolved by reaching consensus. A review-level narrative summary of the risk of bias will also be provided.

To assess the quality of predictors and moderators, we will employ the criteria developed by Pincus et al. [55], van Hoorn et al. [56], and Sun et al. [57], implemented in the study by Knopp et al. [40]. These include the following: (a) predictor and moderator variables are assessed through a validated assessment tool (i.e. published evidence to support adequate psychometric properties of the assessment or instrument used for the target population, non-applicable for non-psychometric variables); (b) predictor and moderator variables are a stratification factor at randomization; (c) fewer than five predictors tested in the model; (d) predictor and moderator variables tested through a priori hypothesis of anticipated effects (as evidenced by a clearly stated hypothesis in the study or a published protocol of the study); (e) for moderator variables, analysis of direct test of interaction between moderator and treatment type is conducted. Each predictor/moderator variable will be evaluated for the above-mentioned criteria with ‘yes’, ‘no’, ‘no sufficient information’, and ‘not applicable’. As advised by van Hoorn and colleagues [56], quality appraisal of predictors and moderators will not yield a total numeric score, but an overall qualitative assessment.

### Data analysis

The final stage of the systematic review will be a data synthesis of the different predictors and moderators of outcomes of psychotherapeutic interventions in YP. Due to our wide inclusion criteria, we anticipate to obtain high heterogeneity across studies; we will thereby conduct a narrative synthesis of extracted data, a method that has been carried out in similar studies [40, 58]. To account for the significant developmental variation within this wide age group, data will be analysed separately for adolescents (12–18 years) and young adults (19–30 years).

Based on previous research, we anticipate that predictor and moderator variables may fall within the following putative, broad categories: (a) sociodemographic variables (gender, age, race, living situation), (b) clinical variables (i.e. baseline symptom severity, onset of mental disorder, comorbid symptomatology), (c) psychological variables (i.e. emotions, personality traits, cognition), and (d) family-related variables (i.e. family environment, relations, communication style). The final grouping and synthesis of predictors and moderators will be determined after we collect the data. Depending on the nature of our findings, our plan is to conduct transdiagnostic and disorder-focused reviews. The reporting of these systematic reviews will be guided by the standards of the Preferred Reporting Items for Systematic Review and Meta-Analysis (PRISMA) 2020 Statement [59].

## Discussion

The study protocol for assessing predictors and moderators of treatment outcome in psychotherapeutic interventions of adolescents and adults aged 12–30 years has been described above. The key strength of this protocol is that it can easily be reproduced and used to facilitate the structuring of other systematic reviews. The protocol was carefully discussed by an interdisciplinary team of early career and senior clinical academics and practitioners, including psychologists, psychiatrists, and a neurologist. They come from diverse psychotherapeutic backgrounds, including cognitive-behavioural, psychodynamic/psychoanalytic, integrative, interpersonal, family therapy/systemic, humanistic/existential, and experiential approaches. The team is composed of researchers from a range of European countries mastering a wide range of languages, which allows a wider selection of studies to be included. The search strategy and protocol have been continuously discussed with experts outside this research team to further improve the quality of the study.

The review is based on state-of-the-art review methods. The PICOS strategy [50] was used to specify the research question and guided the forming of the search strings for this systematic review, and the PubMed and PsycINFO electronic databases were used for the literature search. The review was based on the PRISMA guidelines [49], with initial independent selection of papers by a minimum of two researchers followed by a consensual final selection aided by advice from external experts when required. The Mixed Methods Appraisal Tool will be used for the rating of bias of studies identified and the review was registered within PROSPERO (CRD42020166756).

Given the strengths of this protocol, future studies aiming to develop systematic reviews could use this protocol as a guideline for formulating the research question, search strings, data extraction, critical appraisal, data synthesis, and reporting of results. By extracting a large amount of data from each study, we hope to shed a light on commonly overlooked study characteristics, particularly sociodemographic information and information on the study setting. By including non-randomized and non-controlled studies, the review provides information on a wide range of psychotherapeutic interventions and disorders, which have only been subjected to a limited amount of research. By including self-report measures as well as semi-structured clinical interviews to determine the clinical status of participants, we allowed for a wider more inclusive selection of studies, as for several disorders (e.g. substance use disorders, personality disorders) diagnostic criteria may not always be sensitive enough to capture pathology in youth.

Our overarching aim is to map and synthesize the extant literature, generate hypotheses about predictors and moderators that have not been tested in methodologically robust designs, and offer concrete recommendations for future research based on current methodological limitations. Our study may thereby provide information that can be of interest to several stakeholder groups, such as YP and their families, psychologists, clinicians, school counsellors, and policymakers, as well as providing an impetus for further studies of less explored treatment approaches.

One of the limitations of this study is our age inclusion criterion of 12–30 years will result in several outcome studies for children with typical age below 12 being excluded from the investigation. Such exclusion may be more pronounced for neurodevelopmental disorders, such as autism, attention deficit hyperactivity disorder, oppositional defiant disorder, and conduct disorder. In addition, the wide inclusion criteria employed in our study regarding participants’ age range, the research design of clinical outcome studies, and the clinical diagnostic procedures will likely result in high heterogeneity across studies. We plan to overcome this limitation by conducting subgroup analyses to allow for differentiation of studies based on research designs and other characteristics. Once we gather all relevant predictors and moderators, we will conduct a thematic grouping in ways that will allow us to draw meaningful conclusions. We anticipate that our study will yield multiple systematic reviews, given the wide heterogeneity of our anticipated results.

Despite these limitations, to our knowledge, this is the first systematic attempt to map the current knowledge base on factors influencing the effectiveness of psychotherapeutic interventions in YP.

## Supplementary Information

**Additional file 1.** PRISMA-P 2015 Checklist.

## Data Availability

The dataset is stored in a protected data repository at the University of Copenhagen and can be accessed by requesting it from the first (EV) or the last author (SP). After publishing the systematic reviews, the dataset will be published in a public, open access data repository and will be also uploaded as supplementary information.

## Abbreviations

COST: European Cooperation in Science and Technology
MeSH: Medical Subject Heading
MMAT: Mixed Methods Appraisal Tool
PICOS: The population, intervention, comparison, outcome, and study design (PICOS) strategy
PRISMA: Preferred Reporting Items for Systematic Reviews and Meta-Analyses
TREATme: European Network on Individualised Psychotherapy Treatment of Young People with Mental Disorders
